# Multiple roles of arsenic compounds in phase separation and membraneless organelles formation determine their therapeutic efficacy in tumors

**DOI:** 10.1016/j.jpha.2024.02.011

**Published:** 2024-02-24

**Authors:** Meiyu Qu, Qiangqiang He, Hangyang Bao, Xing Ji, Tingyu Shen, Muhammad Qasim Barkat, Ximei Wu, Ling-Hui Zeng

**Affiliations:** a Department of Pharmacy, Second Hospital of Shanxi Medical University, Taiyuan, 030001, China; b Department of Pharmacology, Zhejiang University School of Medicine, Hangzhou, 310058, China; c Department of Pharmacology, Hangzhou City University School of Medicine, Hangzhou, 310015, China

**Keywords:** Arsenic compounds, Phase separation, Membraneless organelles, PML, Stress granules

## Abstract

Arsenic compounds are widely used for the therapeutic intervention of multiple diseases. Ancient pharmacologists discovered the medicinal utility of these highly toxic substances, and modern pharmacologists have further recognized the specific active ingredients in human diseases. In particular, Arsenic trioxide (ATO), as a main component, has therapeutic effects on various tumors (including leukemia, hepatocellular carcinoma, lung cancer, etc.). However, its toxicity limits its efficacy, and controlling the toxicity has been an important issue. Interestingly, recent evidence has pointed out the pivotal roles of arsenic compounds in phase separation and membraneless organelles formation, which may determine their toxicity and therapeutic efficacy. Here, we summarize the arsenic compounds-regulating phase separation and membraneless organelles formation. We further hypothesize their potential involvement in the therapy and toxicity of arsenic compounds, highlighting potential mechanisms underlying the clinical application of arsenic compounds.

## Highlights

• ATO induces PML-RARa degradation and affects PML nuclear bodies.
• Arsenite promotes nuclear speckles formation.
• Arsenic compounds have dual roles in paraspeckles formation.
• Arsenite brings out stress granules formation.
• Arsenite facilitates processing body formation.

## Graphical abstract

**Figure:**
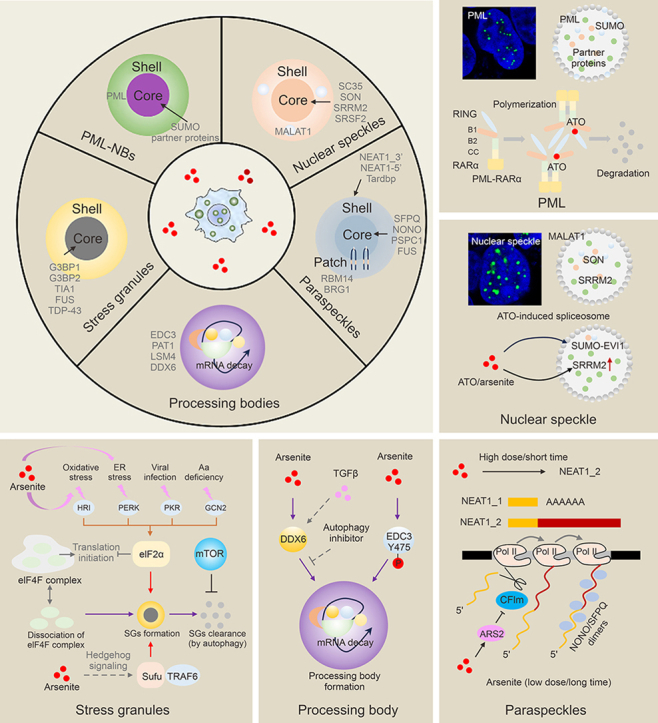

## 1 Introduction

Arsenic is widespread worldwide and contains hundreds of different compounds. Black arsenic and gray arsenic are the two most widely studied allotropes. Arsenic has organic and inorganic forms. Generally, organic arsenic, such as arsenobetaine and arsenocholine, is much less toxic than inorganic arsenic. Moreover, inorganic arsenic has two primary valence forms: trivalent arsenite (As^3+^) and pentavalent arsenate (As^5+^). Trivalent inorganic arsenicals are more toxic than pentavalent inorganic arsenicals. Most arsenic oxides and their salts are poisonous, while hydrogen arsenide is highly poisonous. Arsenic trioxide (ATO) can irritate the eyes, upper respiratory tract, skin, etc. Excess arsenic interferes with normal cellular metabolism, affects respiration and oxidation processes, and causes cellular lesions. Arsenic can also directly damage the walls of small arteries and capillary tubes, acting on the vasodilatory center and leading to increased vascular permeability and causing a decrease in blood volume [1,2].

In addition to toxicity, arsenic compounds are applied to alloy smelting, pigment industry, pesticides, and medicine; their medicinal effects have been discovered for over 2,000 years. In the 1970s, its main component, ATO, was identified as an active ingredient in leukemia. Subsequently, numerous studies found that it has therapeutic effects on various tumors (including breast cancer, hepatoma, lung cancer, etc.). However, its toxicity has been limiting its efficacy, and how to control its toxicity has been a significant issue. Clinically, the combination of all-trans retinoic acid (ATRA) and ATO has been widely applied in acute promyelocytic leukemia (APL) treatment [2,3].

For the pharmacologic mechanism of ATO in APL therapy, promyelocytic leukemia (PML) protein, the core component of PML nuclear body (PML-NB), is the main target of ATO. In recent years, as researchers’ knowledge of phase separation and membraneless organelles has advanced, it has been found that PML forms PML-NB, a membraneless organelle, through phase separation. However, the mechanism of assembly of PML-NB has been unknown. In addition to PML-NB, ATO could induce stress granules (SGs). With the discovery of more membraneless organelles, ATO was found to be widely involved in their formation.

We first search for the clinical application and development of arsenicals through ClinicalTrials and the Pharmacodia database. Next, we briefly describe the principles and pathology of phase separation and membraneless organelles. For the role of arsenicals in phase separation and membraneless organelles formation, we conducted a systematic literature search using the keywords (arsenicals, phase separation, or membraneless organelles) based on PubMed (Medline), Web of Science, and Cochrane Central Database. We elaborate on the concept of phase separation and membraneless organelles and systematically describe how ATO engages in membraneless organelles formation via phase separation, which may be a decisive factor in determining the efficacy and toxicity of ATO, further providing a new theoretical basis for the therapeutic effects of arsenic compounds.

## 2 Arsenic compounds

Since the discovery of arsenic compounds, their roles have been explored for thousands of years, and arsenic compounds play a double-edged role. On the one hand, arsenicals have long been used as poisons due to their toxicity. On the other hand, arsenicals have unique therapeutic properties in tumor treatment.

### 2.1 Toxicities of arsenic compounds

**Fig. 1:**
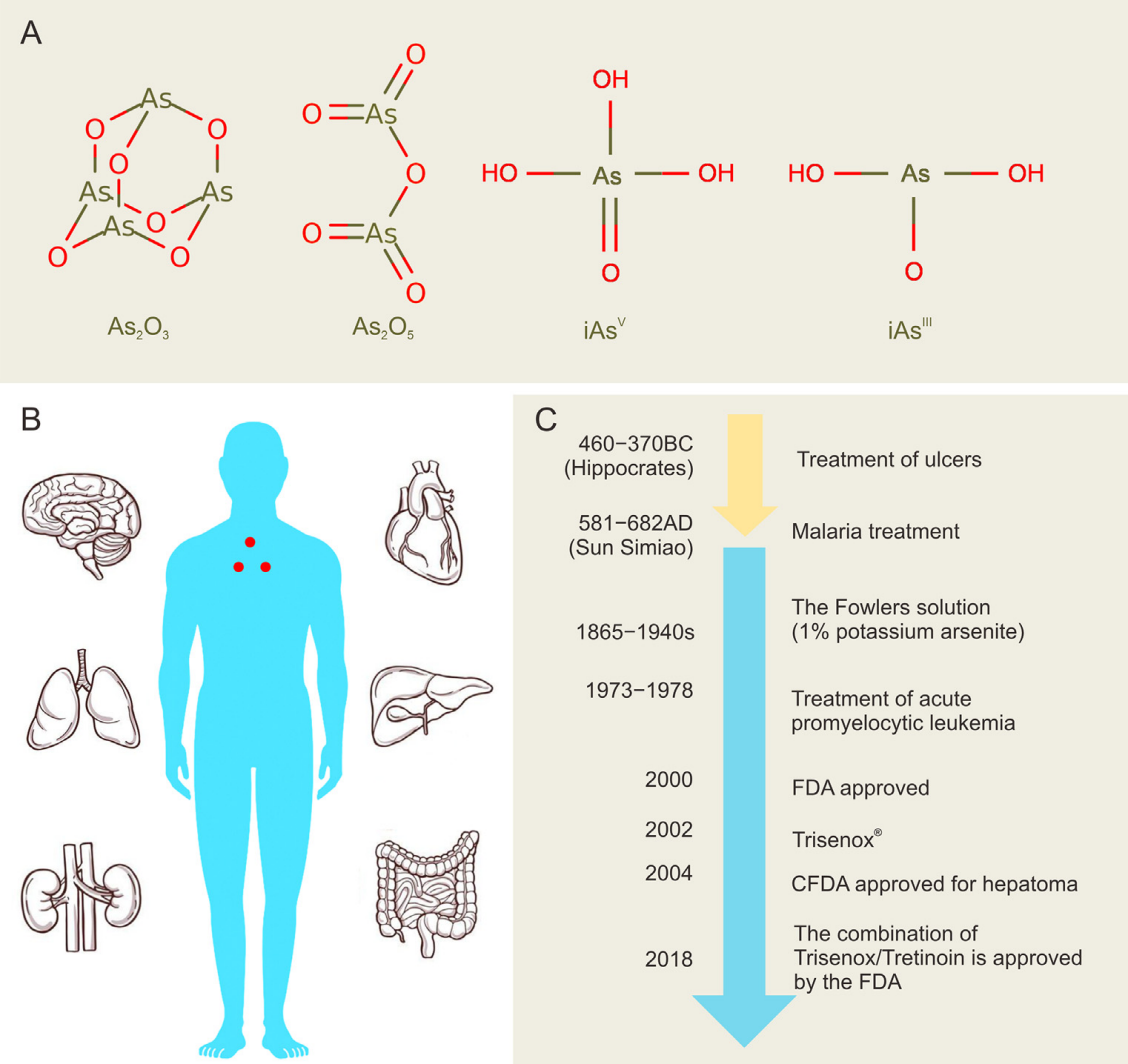
Toxicities and pharmacological effects of arsenic compounds. (A) Structures of different arsenic compounds. Chemical structures of As_2_O_3_ and As_2_O_5_. Two major forms of inorganic arsenic: iAs^V^ and iAs^III^. (B) Toxicities of arsenic compounds in organs. Arsenicals cause neurotoxicity, ischemic heart disease, hepatotoxicity, nephrotoxicity, lung cancer, and bladder tumors. (C) The development of arsenic compounds in human diseases. Arsenic compounds in ancient medicine and its development in modern society. FDA (Food and Drug Administration) and CFDA (China Food and Drug Administration) approve arsenic trioxide (ATO) for acute promyelocytic leukemia (APL). BC: before christ; AD: anno domini.

The toxicity of arsenic is well known. Different arsenicals have different toxicity. The toxicity depends on the organs, cell type, absorption rate, and bioavailability. In humans, acute arsenic poisoning generally manifests throat abnormalities (dryness, pain, and burning sensation) and is accompanied by nausea, abdominal pain, vomiting, and severe diarrhea. Prolonged exposure or ingestion of arsenic compounds leads to the continuous accumulation of arsenite in vital organs, causing skin damage, hepatotoxicity, nephrotoxicity, neurotoxicity, diabetes, atherosclerosis, hypertension, and ischemic heart disease. Moreover, arsenic can induce multiple tumors, including skin, bladder, and lung cancer (Figs. 1A and B).

Skin symptoms are early symptoms in arsenic-poisoned individuals with chronic arsenical exposure and typically develop within a few years with several non-malignant skin disease symptoms, including melanosis, keratosis, hyperkeratosis, and white melanosis. Initially, arsenicals induce melanosis and keratosis; next, white matter melanosis and hyperkeratosis develop, which may eventually progress to transformation into skin cancers [4,5]. In acute arsenic poisoning, arsenics are mainly distributed in the liver and kidney, while long-term arsenic exposure is associated with liver diseases [[6], [7], [8]]. In addition, arsenicals can activate the c-Jun N-terminal kinase (JNK) and p38 mitogen-activated protein kinase (MAPK) pathways and induce apoptosis in hepatocytes [9]. In chicken livers, chronic arsenic exposure leads to ferroptosis through strengthening ferritinophagy [10]. Epidemiological statistics show that nephrotoxic events and chronic kidney disease are correlated with arsenic concentrations [11]. Long-term arsenic exposure can lead to the oncogenic transformation of cancer stem cells. Low concentrations of methylated arsenic can suppress sensory neurons and skeletal muscle, which may explain the low birth weight and neurological abnormality in the population with excessive arsenic levels [12,13]. Trivalent arsenic inhibits the recruitment of GLUT4 to the plasma membrane of adipocytes, which leads to hyperglycemia due to the effect of arsenic and its metabolites through inhibiting insulin-dependent glucose uptake [14,15]. Epidemiological research suggests that arsenic (from food or water) can affect the cardiovascular system, including heart damage, vascular damage, cerebrovascular disease, and hypertension [16,17]. Maternal and childhood exposure to inorganic arsenic is significantly associated with allergic diseases in children [18]. In addition to the above organ toxicity, the International Agency for Research on Cancer (IARC) has classified arsenic as a carcinogen based on epidemiological research [19]. Numerous epidemiological studies have demonstrated a clear relationship between inorganic arsenic exposure and an increased risk of cancer in the skin, lung, prostate, liver, bladder, kidney, and other organs [20,21]. The molecular mechanism of arsenical carcinogenicity is currently unknown, but many studies suggest that it may be due to intracellular signaling, transcription factor activation, and abnormal gene expression.

### 2.2 Clinical applications of arsenic compounds

In addition to being used as a poison, ATO has been applied for thousands of years to treat diseases (Fig. 1C). A Chinese physician, Sun Simiao, used pills containing arsenic to treat malaria. Since the modern era, with the development of medicine, arsenic became a pharmaceutical preparation, among which Thomas Fowler invented Fowler’s solution. Later, Lissauer, a Berlin physician, used Fowler’s solution to improve the condition of a young woman with acute leukemia in 1865. Since then, Fowler’s solution has been used in leukemia and was widely used throughout the 19th century as a remedy for malaria, skin diseases, chorea, edema, rabies, and glandular obstruction. Cytotoxic drugs emerged in the 1940s, while ATO was discontinued after the 1950s due to its toxicity and carcinogenicity.

However, in the 1980s, Zhang et al. [[22], [23]] and Chen et al. [24] reported that intravenous arsenic preparations could alleviate APL patients. In 2000, arsenic acid was commercially available in the US with special approval from the US Food and Drug Administration (FDA). In 2002, the European Commission approved Trisenox® as a rare drug for treating multiple myeloma (MM) and myelodysplastic syndromes (MDS). The China Food and Drug Administration (CFDA) approved ATO for advanced hepatocellular carcinoma in 2004. In 2018, the combination of Trisenox/Tretinoin was approved by the FDA. Currently, the principal strategy of researchers for ATO development is to enhance its efficacy and reduce its toxicity.

The primary role of ATO as one of the first-line clinical agents in APL is to induce the degradation of PML/retinoic acid receptor alpha (RARα) protein [24,25]. APL, an M3 variant of acute myeloid leukemia (AML), expresses a fusion oncogene (a fusion of *PML* gene with *RARα* gene), leading to translation of the PML/RARα chimeric protein, which blocks hematopoietic progenitor cell differentiation and ultimately leads to APL [[26], [27], [28]]. ATO induces PML/RARα degradation through SUMOylation, ubiquitin-proteasome, and autophagy [[29], [30], [31], [32]]. With the application of ATO in the clinical treatment of APL patients, multiple clinical trials have shown that a combination of ATO and ATRA has demonstrated significant efficacy in high-risk APL and pediatric APL patients [33,34].

In addition to its role in APL, ATO has shown therapeutic potential in other hematologic neoplasms, including MDS, lymphoma, lymphoid leukemia, and MM [35,36]. Several trials of patients with MDS on different dosing schedules found moderate clinical efficacy of ATO as a monotherapy for patients with low- and high-risk MDS [[37], [38], [39]]. Similarly, several clinical trials have found that ATO shows a valid therapeutic response in MM cells and moderate clinical efficacy in patients with MM [40,41]. Furthermore, ATO treatment concentrations induced a significant reduction in progenitor cell viability and an increase in apoptosis in non-M3 AML patients compared to other conventional chemotherapeutic agents [42]. Unfortunately, ATO did not observe significant therapeutic effects in non-M3 AML elderly patients [43]. Despite the disappointing results of current clinical trials, its killing effect on malignant tumor cells is still worth exploring. A phase II clinical trial in Asian patients with relapsed or refractory peripheral T-cell lymphoma (r/r PTCL) demonstrated that darinaparsin, an organic arsenical compound of dimethylated arsenic conjugated to glutathione, has anti-tumor activity and acceptable safety profile in patients with r/r PTCL [44].

Moreover, researchers have used various cancer cell lines and animal models to discover that ATO can act against several solid cancers, including hepatocellular carcinoma, ovarian cancers, prostate tumors, and others [[45], [46], [47], [48], [49], [50]]. In hepatocellular carcinoma, ATO can induce tumor cell apoptosis in cell lines. Moreover, it can also evoke the differentiation of cancer stem cells in hepatocellular carcinoma [51,52]. In 2004, the CFDA approved ATO as a single agent in the treatment of hepatocellular carcinoma. Recently, CalliSpheres beads loaded with ATO showed efficacy and safety in transcatheter arterial chemoembolization for unresectable hepatocellular carcinoma patients [53]. Similarly, despite *in vitro* experimental data supporting ATO for treating many of the solid tumors are mentioned above, its efficacy has not been satisfactory in clinical trials. Further studies and clinical trials are needed to explore the therapeutic effects of ATO in solid tumors.

Except for tumors, ATO has demonstrated healing efficacy in other diseases. Chronic graft-versus-host disease (cGVHD) occurs in 20%–50% of recipients after allogeneic hematopoietic stem cell transplantation (allo-HSCT). The first-line therapy for cGVHD is corticosteroids. The combination of ATO and corticosteroids has a high clinical response rate and rapid corticosteroid sparing in cGVHD following previous allo-HSCT [54]. Lupus animal experiments have shown that ATO is effective in systemic lupus erythematosus treatment. Nowadays, a phase II clinical trial has confirmed the safety and efficacy of short-term intravenous ATO in patients with active systemic lupus erythematosus [55].

### 2.3 Advancements of arsenic compounds

Although ATO alone has a tumor-killing effect *in vitro*, due to its inherent toxicity, the current strategy remains to combine it with other drugs, and the combination of ATO with ATRA has become the preferred choice. ATRA (pharmacological concentration) separates PML/RARα from nuclear receptor co-repressors and promotes their association. Their association activates the PML/RARα repressor gene, ultimately triggering APL differentiation. ATRA/ATO combination has a strong synergistic effect on molecular remission in APL patients. Furthermore, primarily hepatocellular carcinoma patients with ATO/locoregional therapy showed better therapeutic responses than locoregional treatment, including higher survival and fewer extrahepatic metastases [56]. In addition, the combination of vitamin C, Venetoclax, Interferon-α, and ATO has better efficacy *in vitro*. It is expected that ATO can combine with more drugs in the future [[57], [58], [59]]. In addition to drug combinations, researchers have developed some novel arsenide nanoformulations to enhance their anti-tumor cell activity, which we will not present here [60,61].

## 3 Membraneless organelles

The toxicity and pharmacological effects of ATO have been relatively well-known over the past decades. However, the cognition of intracellular mechanisms of ATO has also been stagnant over the past decade. Although the mechanism of ATO-induced tumor cell apoptosis is recognized, multiple studies have also pointed out that ATO is involved in intracellular physiological processes such as intracellular signaling transduction, transcriptional activation, and abnormal gene expression. However, a systematic understanding of the role of ATO in cells is still lacking. In recent years, growing evidence suggested that intracellular biochemical reactions do not only depend on membrane organelles (such as mitochondria and endoplasmic reticulum (ER)) but also a large number of membraneless organelles, including PML-NB. Scientists have pioneered the concept of intracellular phase separation and proposed the molecular principle of phase separation driving membraneless organelle formation. These findings provide a new theoretical basis for the role of ATO in the cell.

### 3.1 Phase separation and membraneless organelles formations

**Fig. 2:**
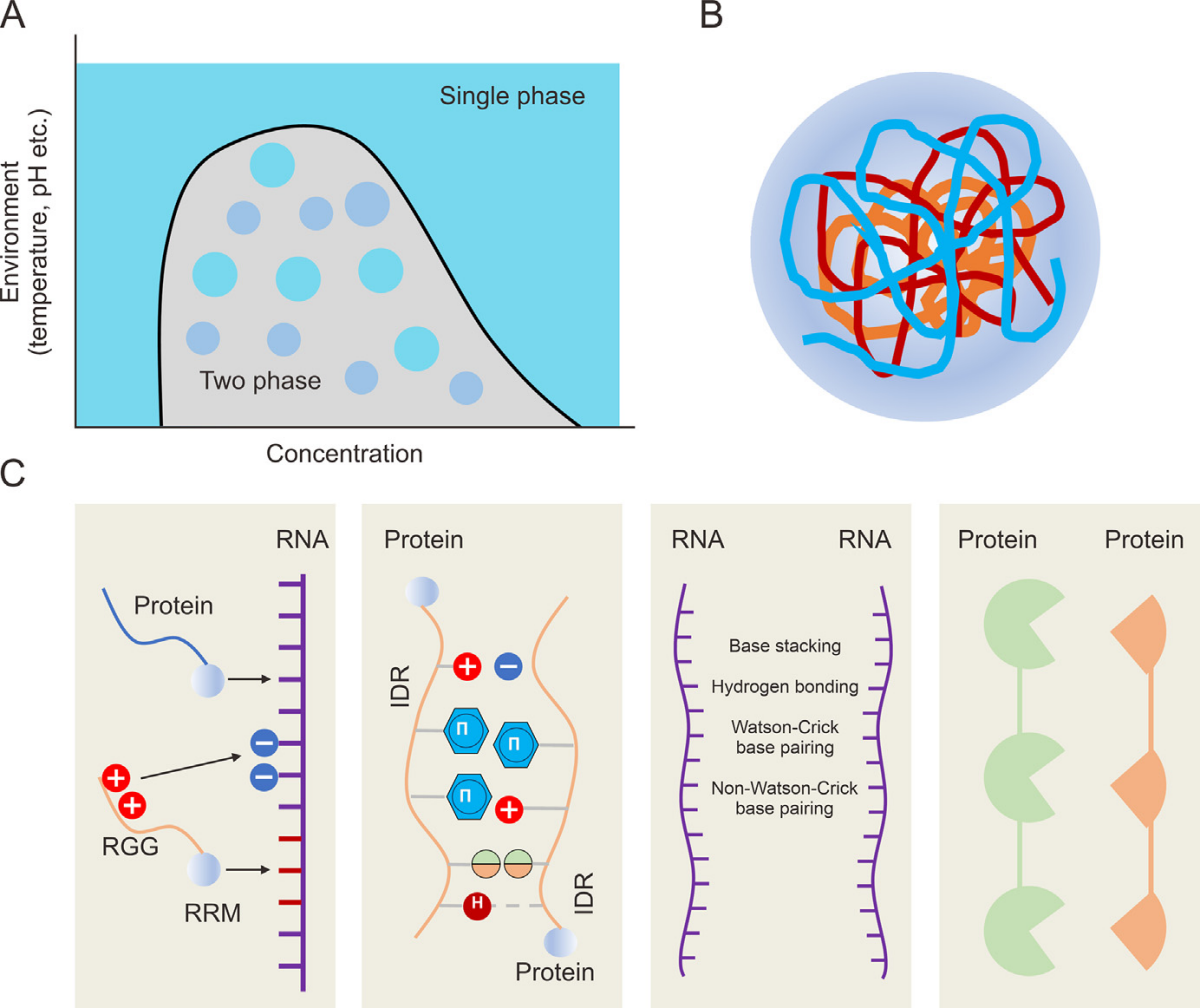
Mechanism of phase separation. (A) Physicochemical principle of phase separation. (B) Phase separation induces condensate formation. (C) The forces driving liquid-liquid phase separation. Multivalent interactions between protein and RNA, protein and protein (intrinsically disordered regions (IDR)), or RNA and RNA. Specific interactions between protein and protein (non-IDR). RGG: arginine-glycine-glycine; RRM: RNA-recognition motifs.

Phase separation was originally a physicochemical concept where binary or multivariate mixtures separate into different phases under certain conditions (Fig. 2A). The oil droplets floating on water is a common phase separation phenomenon. In an experiment to observe the formation of P granules in nematode eggs, Brangwynne et al. [62,63] observed that P granules resemble droplets within the cytoplasm and would collide and fuse. Their research demonstrated intracellular phase separation formation by the characteristics of P granules. Subsequently, Li [64] and Kato et al. [[65], [66]] achieved in vitro reproduction of the *in vivo* phase separation phenomenon by biochemical means, and these studies attracted the attention of the academic community.

Phase separation is a highly dynamic phenomenon. Under certain conditions, various proteins and nucleic acid components in cells or other biological systems spontaneously transform into a low entropy, highly ordered state (Fig. 2B). The molecular basis for phase separation is the formation of “multivalent and low affinity” interactions, such as hydrophobic interactions, and electrostatic interactions. These weak multivalent interactions trigger phase separation between proteins and nucleic acids [67]. Higher valency can form oligomers/polymers at lower saturations. Proteins containing intrinsically disordered regions (IDRs) induce multivalency [64,68]. Multivalent interactions across biomolecules have higher affinity and can assemble into large oligomers or polymers.

Currently, researchers focus on liquid-liquid phase separation (LLPS) driven by IDRs in proteins. IDRs have an unstable structure, which has a broader conformational space and enables protein molecules to form three-dimensional networks with less complex amino acid (AA) sequences in regions containing only a limited AA, such as Gly, Ser, and Gln, and aromatic residues (Phe and Tyr). In addition, electrostatic interactions between oppositely charged residues help to promote LLPS. LAF-1 is a disordered protein, which positively charged Arg/Gly-rich (RGG/RG) structural domain that combines with the negatively charged RNA, effectively promoting the formation of P granules [69]. In addition, several weak interactions, including π-π interactions, cation-π interactions, cation-anion interactions, dipole-dipole interactions, and reversible amyloid interactions, are usually abundant in IDRs/low complexity regions (LCRs) (Fig. 2C). These multivalent weak interactions can induce LLPS [70].

LLPS is dependent on the solution contents and environment, including temperature, biomolecules, salt ion concentration, pH, etc. These biophysical parameters determine the threshold concentration, and when the concentration increases to the solubility limit, the interaction between biomolecules will be stronger than the interface between biomolecules and the solvent, and the solution will tend to LLPS. In short, when the content of these components exceeds a critical concentration (supersaturation), the molecules will spontaneously gather and form a new liquid phase, i.e., LLPS. In brief, the prevailing view on the driving force of phase separation is that LLPS is driven by the non-specific interactions between intrinsically disordered proteins. In addition, some researchers believe that combining “specific interactions between biomolecules” and “non-specific interactions between proteins containing intrinsically disordered sequences” drives phase separation [[71], [72], [73]].

Phase separation *in vivo* explains a long-standing question in biochemistry: How do cells bring together multiple proteins, nucleic acids, small molecules, and other components within the membraneless compartments without the constraints of phospholipid membranes? The LLPS-induced cohesive states can explain this question. These cohesive states are now known as membraneless organelles with physiological functions. We recognize that the membrane structures of organelles such as mitochondria, Golgi apparatus, and ER can assemble a definite class of enzymes and their substrates into a space at a specific time, thus forming a separate compartment that facilitates the reaction to occur and avoids the interference of a large number of extraneous substances in the cytoplasm. However, previous studies have revealed that there are also membraneless structures (such as the nucleolus) that contain various biomolecules and perform physiological functions. The emergence of membraneless organelles has significantly expanded the concept of organelles.

Investigation of the major components of membraneless organelles, such as proteins and RNAs, is continuing. For proteins, researchers can predict the presence of the IDRs*,* a typical structure in proteins that undergo LLPS, based on their protein sequence, chemical valence, and structural features. Hydrophobic amino acids in the primary sequence regulate the concentration of phase separation, while charged amino acids influence the appearance of cohesive states. Thus, we can infer whether the protein undergoes phase separation and the critical concentration of the phase separation. Proteins containing IDRs may also contain RNA-binding structural domains, and RNA will also have protein-binding sequences. The potential of protein phase separation can be predicted by the characteristics of protein and RNA binding. Based on these principles, many researchers have developed different databases for protein phase separation prediction, and these databases also better help researchers (Table 1) [[74], [75], [76], [77], [78], [79], [80], [81], [82], [83], [84], [85], [86]]. Of course, the prediction results of the above databases still need further experimental validation.

**Table 1:** Phase separation databases.

Database	Functions	Websites	Refs.
PhaSepDB	Liquid-liquid phase separation related proteins	http://db.phasep.pro/	[74]
LLPSDB	Proteins undergoing liquid-liquid phase separation *in vitro*	http://bio-comp.ucas.ac.cn/llpsdb http://bio-comp.org.cn/llpsdb	[75]
DisPhaseDB	Diseases related variations in liquid-liquid phase separation proteins	http://disphasedb.leloir.org.ar	[76]
RPS	RNAs involved in liquid-liquid phase separation	http://rps.renlab.org	[77]
PhaSePro	Proteins driving liquid-liquid phase separation	https://phasepro.elte.hu	[78]
RNAPhaSep	A resource of RNAs undergoing phase separation	http://www.rnaphasep.cn	[79]
DrLLPS	Liquid-liquid phase separation in eukaryotes	http://llps.biocuckoo.cn/	[80]
RNAgranuleDB	A consensus stress granule proteome through literature curation	http://rnagranuledb.lunenfeld.ca/	[81]
MobiDB	Predictions and annotations for intrinsically disordered proteins	https://mobidb.org/	[82]
MloDisDB	Database of the relations between membraneless organelles and diseases	http://mlodis.phasep.pro/	[83]
PRALINE	Protein and RNA human single nucleotide variants in condensates	http://praline.tartaglialab.com	[84]
Disprot	The major repository of manually curated annotations of intrinsically disordered proteins and regions	https://disprot.org	[85]
FoldUnfold	Prediction of disordered regions in protein chain	http://bioinfo.protres.ru/ogu/	[86]

Currently, only a few quantitative methods have been applied in the study of LLPS, among which the representative one is the fluorescence recovery after photobleaching (FRAP), which characterizes the molecular dynamics in the liquid-liquid separation phase [87]. Theoretically, the molecules in the liquid system have large motility, and the surrounding unbleached fluorescent molecules will enter the bleached area due to diffusion, thus allowing the fluorescence to be gradually recovered. However, the recovery time of FRAP reported in different studies varies due to multiple factors such as concentration, binding and diffusion, and instrumentation. New technical tools are emerging to identify LLPS accurately in the cell. Advances in optical microscopy and spectroscopy have made it possible to quantify and compare the absolute abundance of proteins in LLPS. Brangwynne's team [88] developed a range of tools to investigate membraneless organelles, optoDroplet was developed in 2016, which provides unprecedented access to manipulate and understand the chemical reactions that allow membraneless organelles to function. In 2018, their team formulated another tool called Corelets; they used it to quantitatively characterize the concentration of proteins in cells that promote phase separation. The Corelets use photosensitive proteins that have been genetically modified to undergo morphological changes and alter their behavior when exposed to light. The researchers can use this technique to trigger phase separation in different cellular regions by changing several parameters [89]. In addition, Brangwynne’s team [90] developed a tool called CasDrop, an optogenetic tool based on Clustered regularly interspaced short palindromic repeats (CRISPR)-CRISPR-associated protein (Cas)9 gene editing technology that determines the location of specific genes in a cell. Once it was activated by light, other proteins bind to specific genes, leading to phase separation locally, and formulating tiny droplets on chromatin. These tools can help researchers to investigate membraneless organelle formation. In addition, single-molecule tracking techniques will replace FRAP as a better way to study molecular dynamics properties. Experimental models of LLPS regulated by light or small molecules have emerged.

### 3.2 Membraneless organelles and human diseases

**Fig. 3:**
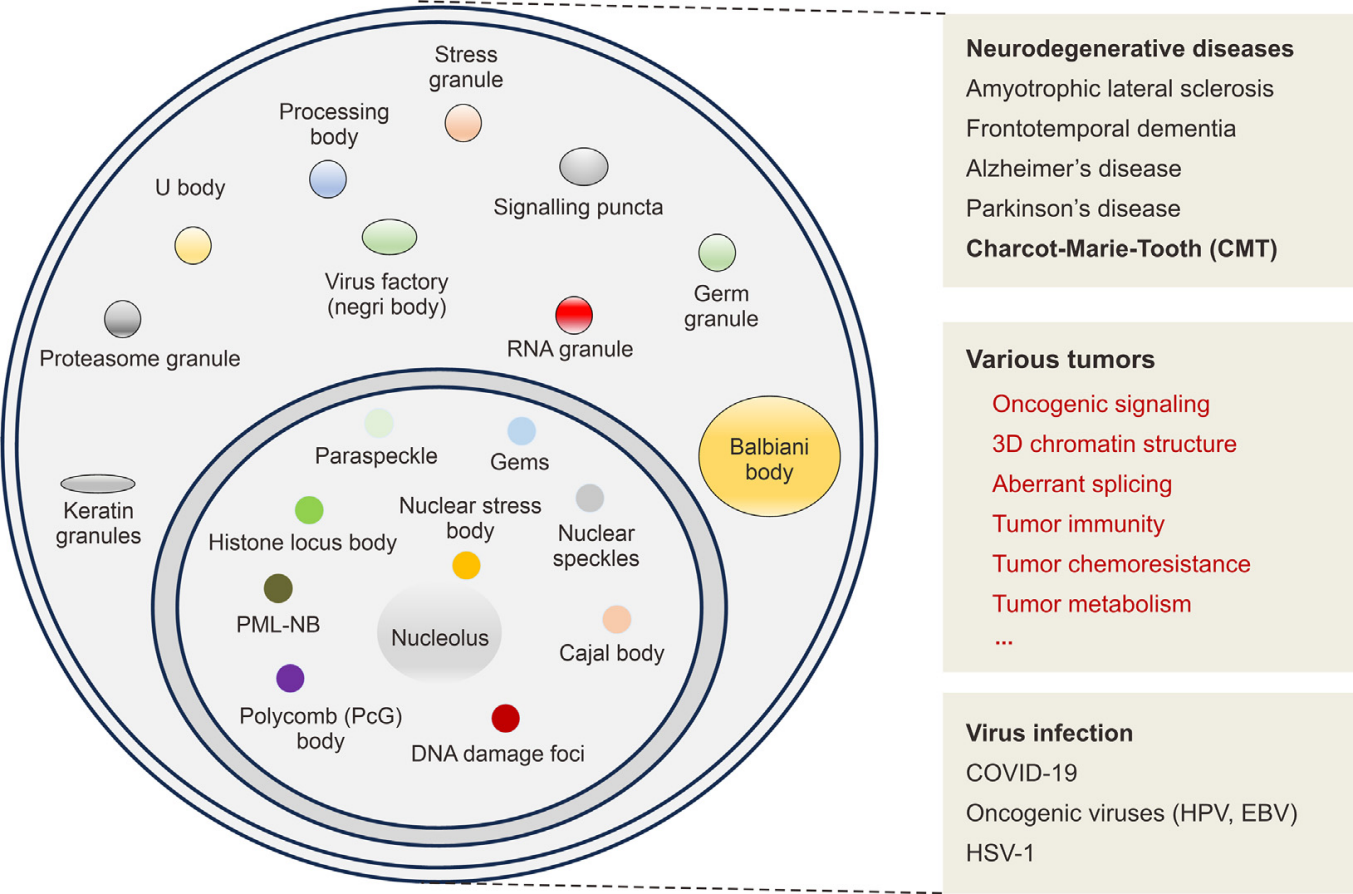
Phase separation and membraneless organelles. Phase separation induces membraneless organelles formation. The membraneless organelles identified include stress granules (SGs), processing bodies, promyelocytic leukemia (PML) nuclear bodies, etc. Phase separation is closely related to neurodegenerative diseases, multiple tumors, and virus infections (corona virus disease 2019 (COVID-19)), human papillomavirus (HPV), Epstein-Barr virus (EBV), and herpes simplex virus type 1 (HSV-1).

Nowadays, researchers realize that membraneless organelles formed by phase separation are widely present in different cells. The membraneless organelles identified so far include stress granule, processing body (P-body), Tiger domain, PML nuclear body, Cajal body, nuclear speckles (NSs), paraspeckle (PS), etc. Each of these different membraneless organelles has different composition and physiological functions (Fig. 3), and they are the executors of spatial and temporal coordination of various biological activities in the cell, including chromatin organization, genome stability, immune response, and signal pathways. The physiological functions of phase separations have been described in many reviews, and we will not go into detail here. Conversely, abnormal phase separation processes *in vivo* can lead to a range of pathological responses, and the relevance of diverse membraneless organelles to disease has been well established.

#### 3.2.1 Membraneless organelles and tumorigenesis

With the progressive understanding of the components of membraneless organelle**s**, the relevance of LLPS to tumor biology is becoming clear. Similar to neurodegenerative diseases, mutations in the main factors of membraneless organelles alter the physical properties of membraneless organelles, leading to the acquisition of toxic membraneless organelles or the loss of functions of these factors through abnormal processes such as protein aggregation.

The membraneless organelles components include oncogenic genes and tumor suppressors. TP53 is one of the most common tumor suppressors and is the most frequently mutated gene in human cancers, occurring in more than 50% of all tumors [91,92]. Many TP53 mutations cause the loss of its functions and enhance its oncogenic effect. In addition, TP53 mutations can also induce protein aggregates [[93], [94], [95]]. The isolated structural domain of TP53 can form amyloid at low pH and progressively higher temperature and pressure [96,97]. Speckle-type POZ protein (SPOP), another tumor suppressor, is frequently mutated in solid tumors, including breast, colon, gastric, kidney, and prostate cancers [[98], [99], [100]]. Tumor-related mutations in SPOP are found in the substrate-binding meprin and tumor necrosis factor receptor-associated factor (TRAF) homology (MATH) domain. SPOP and DAXX co-localize in liquid nuclear organelles and undergo phase separation *in vivo*. SPOP mutants disrupt LLPS and DAXX ubiquitination [101]. KRAS is one of the most commonly mutated oncogenes in cancer, such as pancreatic and colon cancers [102,103]. Most of these point mutations alter amino acids G12, G13, or Q61, resulting in loss of GTPase activity [104]. KRAS mutants produce prostaglandins in response to stress stimuli. KRAS mutant cells secrete prostaglandins to neighboring cells, enhancing the formation and resistance of SGs in KRAS Wilms tumor (WT) cells. Promoted SGs are cytoprotective against stressful stimuli [105,106]. Both activated and inactivated mutations in Src homology 2-containing protein tyrosine phosphatase 2 (SHP2) can trigger oncogene signaling by altering SHP2 conformation. The condensate formed by activating SHP2 mutants induces overactivation of the RAS-extracellular signal-regulated kinase (ERK) pathway through its inherently high protein tyrosine phosphatase activity [107]. The phase separation of these oncogenes or tumor suppressor genes caused by mutations activates a series of oncogenic signals that promote tumorigenesis.

Secondly, phase separation can also drive tumorigenesis by altering chromatin spatial structure. Chromosomal translocation-mediated formation of fusion proteins promotes cancer development, including leukemia [108]. These oncogenic fusion proteins often form membraneless organelles that control gene expression via multiple molecular mechanisms. For example, the fusion oncoprotein NUP98-HOXA9 (the IDRs (FG repeat) of NUP98 and the DNA-binding structural domain of HOXA9) drives abnormal remote chromatin interactions via LLPS, leading to acute myeloid leukemia [108]. Loss of LLPS by ubiquitously transcribed tetratricopeptide repeat on chromosome X (UTX) mutants also disrupts chromatin regulation. UTX is frequently mutated in cancer, with the most common changes being nonsense mutations near the beginning of the core IDR. These mutant UTX variants (UTX∗) fail to segregate from MLL4 and p300, leading to dysregulated chromatin state and tumorigenesis [109].

Thirdly, a large number of aberrant splicing promotes tumorigenesis. For example, SHH-medulloblastoma (SHH-MB) is accompanied by aberrant splicing of key regulatory molecules of the SHH signaling pathway [110]. However, mRNA splicing is closely related to the membraneless organelles (paraspeckle and nuclear speckle). It has been suggested that paraspeckle formation is associated with lower prognostic survival [111]. A previous study found that the intranuclear protein AKAP95 can regulate gene expression, especially mRNA splicing. AKAP95 directly regulates mRNA splicing of some cancer genes, thereby supporting cancer cell growth [112]. These results suggest that membraneless organelles formed by LLPS in the nucleus may promote tumorigenesis by regulating alternative splicing.

Then, LLPS is also involved in tumor immunity; the KAT8-IRF1 condensate promotes programmed death-ligand 1 (PD-L1) expression in tumor cells. Specific peptides can block the formation of this condensate and enhance anti-tumor immunity [113]. The cyclic guanosine monophosphate (GMP)-adenosine monophosphate (AMP) (cGAMP) can bind to the ER transmembrane interferon gene receptor-stimulating factor (STING), which then travels to the Golgi apparatus and activates TANK-binding-kinase 1/inhibitor of κB kinase (TBK1/IKK), triggering the production of type I interferons and pro-inflammatory cytokine production. Interestingly, LLPS forms condensate containing ER that buffers cGAMP and suppresses immune responses [114].

Next, membraneless organelles affect the pharmacodynamic properties of antitumor drugs, and stress granule assembly is associated with cancer cell survival in chemotherapy, indicating that SGs enhance chemoresistance [105,[115], [116], [117]]. LLPS of androgen receptor (AR) also plays a significant role in the mechanism of anti-androgen resistance in prostate cancer. The androgen dihydrotestosterone (DHT) induces LLPS of AR into the nucleus and forms condensates that serve as active transcription factories. LLPS of AR is directly related to the transcriptional activity and anti-androgen sensitivity of the AR. The AR antagonist enzalutamide eliminated the formation of condensates by LLPS of the wild-type AR but did not eliminate the formation of condensates by the drug-resistant mutant AR (AR F877L/T878A). This study also developed LLPS inhibitors targeting the AR, which were able to inhibit the transcriptional activity of the AR and effectively inhibit the tumor growth of prostate cancer cells expressing drug-resistant mutant AR *in vivo* [118]. The link between LLPS and drug resistance has received increasing attention.

Overall, malignant cells with genomic mutations have significant roles in tumorigenesis via LLPS-mediated multiple biological processes, including transcription, DNA damage/repair, and chromatin changes. The mutations of tumor suppressors and oncogenes lead to aberrant cellular activities, including proliferation, angiogenesis, invasion, metastasis, and resistance to cell death [67]. Aberrant LLPS can also affect epigenetic regulation, which has also been implicated in cancer development and progression. In addition, membraneless organelles also influence the efficacy of oncology drugs.

Phase separation and membraneless organelles are tightly controlled by cellular protein degradation and molecular chaperone mechanisms. Searching for drugs that upregulate these pathways or generate potent engineered depolymerization molecules that can antagonize pathological phase separation will be significant to drug development targeting phase separation. Interestingly, more and more researchers found the multiple roles of arsenicals in regulating phase separation and related pathways.

#### 3.2.2 Membraneless organelles and other diseases

Except for tumors, protein aggregation is a significant biomarker of numerous neurodegenerative diseases, such as amyotrophic lateral sclerosis (ALS), frontotemporal dementia (FTD), Alzheimer’s disease (AD), and Parkinson’s disease (PD) [119]. The TAR-DNA binding protein 43 (TDP-43) shifts from a reversible dynamic LLPS to an irreversible aggregated state fused in sarcoma (FUS), tau, and α-synuclein, respectively, in aggregates were found in damaged neurons of ALS, FTD, AD, and PD patients, respectively [120,121]. Similarly, in COVID-19, the novel coronavirus invades the host cell and could release RNA genome for numerous proteins translation, including nonstructural and structural proteins [122,123]. COVID-19 has four main structural proteins, including membrane (M), envelope (E), nucleocapsid (N), and spike-in (S) proteins. Among them, the N protein is involved in the transcription, replication, and packaging [72,124]. New evidence suggests that N proteins achieve these functions through phase separation [125,126]. Furthermore, nucleoli, Cajal bodies, and PML-NBs are closely related to various malignant cells. Here, we focus on the relevance of phase separation to tumors.

## 4 The emerging roles of arsenic compounds in membraneless organelles

ATO, as an antitumor agent, acts in APL by targeting the PML protein, which is a core component of PML-NBs; Similarly, it has a regulatory effect on PML-NBs. However, the role of ATO in other membraneless organelles remains unknown. Here, we try to systematically elucidate its functions on membraneless organelles (Table 2) [25,[127], [128], [129], [130], [131], [132], [133], [134], [135], [136], [137], [138], [139], [140], [141], [142], [143], [144], [145], [146], [147], [148], [149], [150], [151], [152]].

**Table 2:** Effects of arsenic compounds on membraneless organelles.

Organelles types	Diseases research subject	Functions	Refs.
PML nuclear bodies	Leukemia	Degrades promyelocytic leukemia (PML)/retinoic acid receptor alpha (RARα) oncoprotein and controls PML nuclear bodies assembly.	[25,[127], [128], [129], [130]]
	Human adenoviruses	Arsenite blocks virus expression and replication by reducing the number and integrity of PML nuclear bodies.	[131]
	Pediatric glioma	H3.3 K27 M patient-derived glioma cells have abnormalities in PML nuclear bodies and are sensitive to As_2_O_3_.	[132]
	Neuroblastoma	As_2_O_3_ induces a rapid assembly of PML nuclear bodies in an extracellular signal-regulated kinase (ERK)-dependent manner.	[133]
	Liver/Cardiac/Lung fibrosis	As_2_O_3_ was able to significantly trigger PML SUMOylation and the formation of PML nuclear bodies.	[[134], [135], [136]]
Nuclear speckle	Human keratinocytes	Splice regulators were induced upon chronic arsenic exposure.	[137]
	HEK293T cells	As_2_O_3_ enhances the co-localization of ecotropic virus integration site1 (EVI1) and SUMO1 in nuclear bodies.	[138]
Paraspeckle	Osteosarcoma U2OS cells	Arsenic resistance protein 2 (ARS2) suppresses the formation of paraspeckles.	[139]
	Neurodegenerative diseases	Short-term arsenite stimulation can promote paraspeckle formation.	[140]
Stress granule	Neurodegenerative diseases	Arsenic trioxide-induced stress granule formation.	[[141], [142], [143]]
	Lung cancer		[144]
	Glioblastoma		[145]
	Prostate cancer		[146]
	Cervical cancer		[147,148]
	Leukemia		[149]
Processing body	Osteosarcoma (U2OS cells)	Stress-induced processing body formation.	[150,151]
	Cervical cancer (Hela cells)		[152]

### 4.1 Arsenic compounds and PML nuclear bodies

PML-NBs are spherical membraneless organelles that are 0.1–1 μm in diameter. It is a laminar structure containing a 100 nm thick shell [153,154]. This shell is formed by multivalent interactions of small ubiquitin-like modifiers (SUMO) proteins bound to PML with the SUMO interaction motif (SIM) of PML-interacting proteins. It surrounds a core filled with many different proteins. Until now, over 170 proteins have been identified as constituent or transient PML-NBs components. Numerous researches suggested that PML-NBs are involved in various cellular functions, including stem cell self-renewal, transcriptional regulation, DNA damage responses, cell fate, and so on [155].

The PML protein is encoded by the *PML* gene, which was first discovered in APL patients and encodes the N-terminal part of the PML-RARα fusion oncoprotein. PML protein was initially identified as a fusion partner of RARα in APL and is a tumor suppressor [156,157]. PML-RARα fusion is considered a significant driver of APL and is observed in most APL patients. Mechanistically, PML inhibits tumor cell proliferation, migration, and invasion; it promotes apoptosis and senescence. PML also regulates neovascularization, tumor metabolism, and tumor stem cell maintenance [[158], [159], [160], [161]]. ATO and ATRA can directly target PML-RARα, leading to the cure of 95% of APL patients in the past 30 years.

**Fig. 4:**
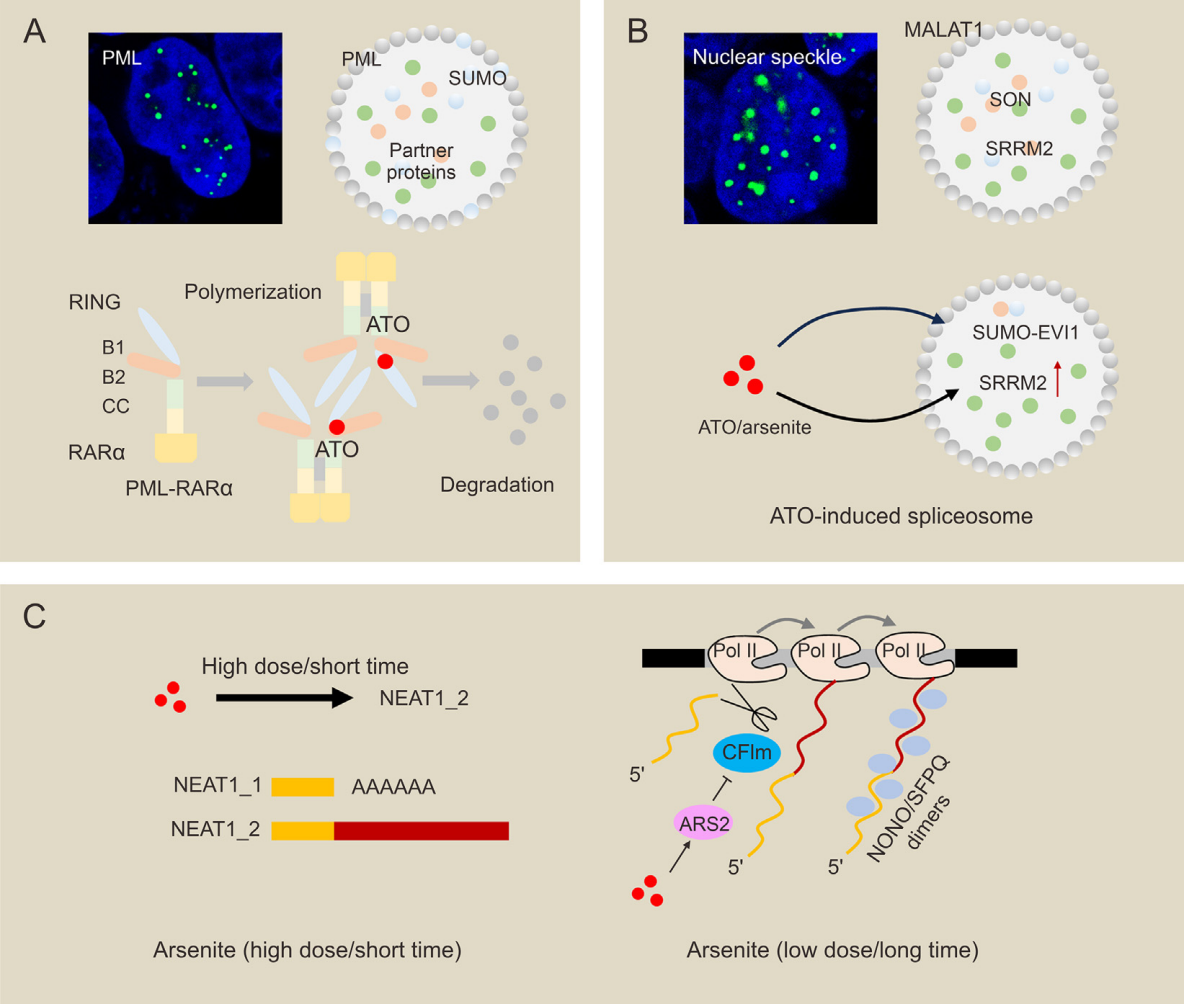
Arsenic compounds and membraneless organelles formations in the nucleus. (A) Arsenic trioxide (ATO) induces promyelocytic leukemia (PML)-retinoic acid receptor alpha (RARα) degradation and disrupts PML nuclear bodies. (B) The mechanism of arsenite-induced nuclear speckles formation. Chronic arsenic exposure promotes nuclear speckles formation via inducing splicing factor serine/arginine repeat motif 2 (SRRM2). (C) The dual roles of arsenic compounds in paraspeckles formation. Short-term arsenite stimulation can promote paraspeckle formation via a significant increase in the levels of total NEAT1 RNA and lncRNA isoform NEAT1_2. ATO treatment can also inhibit paraspeckles by inducing arsenic resistance protein 2 (ARS2) expression. MALAT1: metastasis-associated lung adenocarcinoma transcript 1; SON: SON DNA and RNA binding protein; EVI1: ecotropic virus integration site1; CFIm: mammalian cleavage factor I; SFPQ: splicing factor, proline- and glutamine-rich; NONO: non-POU domain-containing octamer-binding protein.

PML shows extraordinarily dynamic activities, which can translocate from the nucleoplasm to PML-NBs [153,162]. In APL, PML-NBs are grossly impaired by PML-RARα fusions. ATO can enhance PML SUMOylation, leading to the self-destruction of oncogenic fusions through the proteasomal pathway. In addition, ATO-induced PML-RARα degradation also contributes to the restoration of PML-NBs assembly and apoptosis [25,163]. PML-NBs are also significant regulators of ATO-targeted therapy. Based on the frequent observation of chemically modified ATO and PML-RARα mutants in ATO-resistant APL patients, the RBCC structural domain may be the ATO binding site [164]. Recent research found that PML-RARα mutations (L73E and F52E/F54E) exhibit less NB recapitulation, SUMOylation, and terminal differentiation than PML-RARα (WT). These evidence support the hypothesis that PML oligomerization may initiate ATO-PML interactions (Fig. 4A) [165]. In addition to APL, ATO blocks viral expression and replication by reducing the number and integrity of PML-NBs (important subnuclear structures for hepatitis A virus (HAV) replication) [131].

### 4.2 Arsenic compounds and nuclear speckles

Except for PML-NBs, ATO can improve the SUMOylation of Ecotropic virus integration site1 (EVI1) and enhance the co-localization of EVI1 and SUMO1 in nuclear bodies different from PML-NBs [138]. EVI1 is mostly present in a diffuse form, with about 5%–10% of cells showing nuclear speckles [166]. Pearson’s coefficient showed a positive association between EVI1 and SUMO1 in nuclear speckles (Fig. 4B). Nuclear speckles were probed as several tens of nuclear foci ranging in size from 0.3 to 3 μm, in which various snRNPs, splicing factors, and RNAs containing poly(A)-tailed (poly(A)+) Pol II transcripts were localized [167]. The nuclear speckles also include a multilayered structure, with a shell highly enriched by snRNA and metastasis-associated lung adenocarcinoma transcript 1 (MALAT1) lncRNAs, the encapsulated core containing the splicing factor serine/arginine repeat motif 2 (SRRM2) and the SON DNA and RNA binding protein (SON). Proteomic analysis revealed hundreds of proteins enriched in nuclear speckles involved in various stages of RNA processing and nuclear export. These proteins are also widely present in the nucleoplasm [168]. The nuclear speckles exhibit liquid condensation, which is dependent on LLPS. SC35 is the most commonly localized marker of nuclear speckles and is proposed for biochemically purified spliceosomes [169]. Similarly, serine/arginine-rich splicing factor 2 (SRSF2) can be used as a marker for nuclear speckles [170]. Recent studies suggested that SON and SRRM2 are essential for nuclear speckles formation. The long IDR in SRRM2 passively isolates other condensation-prone RBPs, inducing phase separation and assembly of nuclear speckles [171,172]. Nuclear speckles are dynamic structures whose size and shape can vary by cell type and depend on many factors, including cellular ATP levels, phosphorylation of multiple proteins, transcription of stress-activated genes, SWI/SNF chromatin remodeling, and transcription and splicing of RNA polymerase II [173]. The function of nuclear speckles remains unclear. What is clear to the researchers is that the formation of nuclear speckles is closely related to transcription and RNA processing. RNAPII binds transcript synthesis to DNA templates and regulatory proteins; It can also combine RNA maturation and export [174]. The genome-wide analysis suggested that a significant portion of active genes are enriched near the nuclear speckles [175]. In brief, nuclear speckles promote transcription and co-transcriptional processes, including speckle-attached genes splicing in the phase separation space.

Numerous previous studies have shown that arsenide exposure induces a range of abnormal alternative splicing. For example, States et al. showed that acute arsenide exposure affects the splicing model of ZRANB2 protein in HaCaT cells (human keratinocytes) [176]. Recent studies found that RNA metabolism and splicing regulatory pathways are significantly enriched in HaCaT Cells exposed to 100 nM NaAsO2 for 7 weeks. It also uncovered that chronic arsenic exposure induces SRRM2, which may be responsible for arsenic-induced nuclear speckles (Fig. 4B) [137]. The generation of NSs seems to explain selective splicing as a mechanism of arsenic-induced toxicity and carcinogenicity.

### 4.3 Arsenic compounds and paraspeckle

Distinct from nuclear speckles, arsenic compounds may play a dual role in paraspeckle formation. Recent studies have suggested that PS formation relies on recruiting specific protein components by seed sequences of specific lncRNAs, whereby protein-RNA interactions occur, resulting in locally high concentrations at a particular location. Once bound to their backbone lncRNAs, these proteins undergo oligomerization and recruit other LCD-containing proteins to mediate the process of LLPS and form paraspeckes. Paraspeckle is a membraneless organelle of variable size and uneven distribution, with a size of about 0.2–1 μm. It was first identified in HeLa cells, and subsequent studies have shown that it is widely distributed in mammalian tissues and cells, with approximately 10–30 present in each nucleus [177]. In 2002, a study using mass spectrometry to analyze the proteomics of purified nucleoli identified a new protein that is not enriched in the nucleoli but diffusely distributed in the nucleoplasm. This protein is concentrated in several subnuclear spots, which were localized near but distinct from nuclear speckles between chromatin, hence the name paraspeckle for these spots. The new protein localized to these structures was subsequently named paraspeckle protein 1 (PSPC1) [178]. Although the initial PS was thought to be a PSPC1-enriched region, PSPC1 knockdown doesn’t affect PS formation [179], while two proteins, NONO and SFPS, are essential for PS formation. Several proteins are also significant for PS formation, including HNRNPK, DAZAP, FUS, RBM14, HNENPH, etc. [180] In addition, NEAT1 is a mammalian-specific lncRNA that is a structural component of paraspeckle, and deletion of NEAT1 leads to PS disassembly [181]. There is a limited understanding of the functions of PS, and the main functions known are related to its protein and RNA components. In ALS, PS is associated with many cytoplasmic aggregates of PLD-containing proteins, which are usually caused by mutations in PLD-containing proteins. The relationship between PS and tumors varies significantly depending on the cancer type. PS appears to have oncogenic effects in several tumors. Hypoxia leads to an increase in NEAT1_2 transcription through hypoxia-inducible factors, increasing the number of PS in breast cancer cell lines [111]. In prostate cancer, elevated levels of NEAT1_1 and NEAT1_2 correlate with advanced-stage of disease [182]. In skin fibroblasts, p53 induces paraspeckle when preneoplastic cells transform into tumors in hereditary and localized skin cancer mouse models, but NEAT1 deletion prevents this transformation, suggesting that NEAT1 is a potent oncogene [183]. A deeper understanding of PS still needs to start from the function of its various protein components.

Wang et al. found that arsenite (100 μM, 2 h) induces a significant increase in the levels of total NEAT1 RNA and lncRNA isoform NEAT1_2 in the neurons of excitable mice, suggesting that short-term AS stimulation can promote PS formation (Fig. 4C) [140]. However, prolonged arsenical treatment (10 μM, 6 h) may reverse this process, which is dependent on arsenic resistance protein 2 (ARS2). The 3′-terminal processing and stability of NEAT1 is regulated by ARS2. ARS2 knockdown inhibits the association between NEAT1 and mammalian cleavage factor I (CFIm), resulting in a shorter NEAT1_1 isoform. In addition, ARS2 knockdown leads to preferential stabilization of NEAT1_2. As a result, NEAT1_2 levels were significantly elevated in ARS2 knockdown cells, inducing the number of paraspeckle. These results revealed that ARS2 has a repressive role in NEAT1_2 expression and subsequent paraspeckle formation. ARS2, a highly conserved protein, was shown to bind to the mRNA 5′-end cap-binding complex (CBC) and regulate RNA synthesis, shearing, translation, and other functions with other enzymes or proteins. Previous studies have shown that short-term arsenite does not affect ARS2, while prolonged treatment induces ARS2 expression and thus inhibits paraspeckle [139].

Overall, ATO plays different roles in PS formation depending on the timing. Interestingly, clinical samples show that ARS2 is highly expressed in AML samples and correlates significantly with patient prognosis. However, it has been noted that patients with relapsed APL have lower levels of ARS2 expression than the remaining consecutive CR patients and that low expression of the ARS2 gene may adversely affect treatment outcomes. It implies that high ARS2 expression may make AML cells more sensitive to ATO treatment and that prolonged induction of ARS2 expression by ATO may prevent their relapse and thus improve the efficacy of ATO [184]. With ARS2 expression induced by ATO treatment, PS formation may be inhibited, which may be a significant basis for assessing the treatment effect (Fig. 4C).

### 4.4 Arsenic compounds and SGs

In addition to membraneless organelles in the nucleus, arsenite also affects some membraneless organelles in the cytoplasm. Early on, researchers found that arsenite induces the formation of SGs. SGs are membraneless organelles formed within the cytoplasm of eukaryotic cells through LLPS assembly in response to external environmental stresses (oxidative stress, altered osmotic pressure, viral infection, etc.). SGs are composed of mRNA, small ribosomal subunits, translation initiation factors, RNA-binding proteins, etc. [185]. These components constitute a low-density shell and a dense core, respectively; the shell can exchange substances with the cytoplasm dynamically, while the core is less dynamic [186]. Under stress conditions, translation initiation is blocked and accompanied by the disassembly of polyribosomes and aggregation of mRNA; then, mRNA interacts with SG nucleating protein G3BP1 and assembles the core of SG by LLPS. The core matures with the addition of new RNA-binding proteins (TIA1, FUS, TDP-43, etc.) and mRNA, followed by further recruitment of proteins through protein-protein interactions and the fusion of small particles, thus assembling into larger particles visible under the microscope [187]. There are two main pathways of translation initiation blockage caused by SG formation under different stress conditions: the eukaryotic translation initiation factor 2 subunit alpha (eIF2α) phosphorylation pathway and the eukaryotic translation initiation factor 4F (eIF4F) complex disassembly pathway. eIF2α subunit at Ser51 is phosphorylated, leading to the disassembly of the ternary complex and the blockage of translation initiation. And kinases such as general control nonderepressible 2 (GCN2), protein kinase R (PKR), protein kinase RNA-like ER kinase (PERK), and heme-regulated inhibitor (HRI) can participate in the phosphorylation of eIF2α [[188], [189], [190]]. eIF4F complex assembly on the mRNA cap is one of the checkpoints for translation initiation. The assembly process of the eIF4F complex is mainly under the strict control of the target protein of rapamycin, mechanistic target of rapamycin (mTOR) [191]. SG is a reversible structure that temporarily exists under stress, assembles when it encounters stress, and rapidly disassembles when pressure is relieved. SG can generally be cleared by two pathways: chaperone-mediated decomposition and autophagy. Generally cleared mainly by non-autophagic pathways mediated by chaperone proteins, various heat shock proteins associated with SG disassembly have been identified, such as HSP40, HSP70, HSP90, and HSP104. Among these proteins, a monitoring system involving the ternary chaperone complex (HSP8-BAG3-HSP70) regulates SG disassembly and clearance of misfolded proteins to maintain SG integrity and dynamics [192]. However, SG forms stable irreversible amyloid aggregates in some cases, and these persistent SGs are cleared via autophagy-dependent pathways [193].

The association of SGs with neurodegenerative diseases does not need to be described any further. The tumor microenvironment exhibits hypoxia, nutrient deficiencies, and an imbalance in the redox state, which in themselves can be environmental stresses that can directly induce the assembly of SGs. SGs contribute to the adaptation of tumor cells to external stresses. Moreover, SG induces tumorigenesis, proliferation, metastasis, and drug resistance [194].

**Fig. 5:**
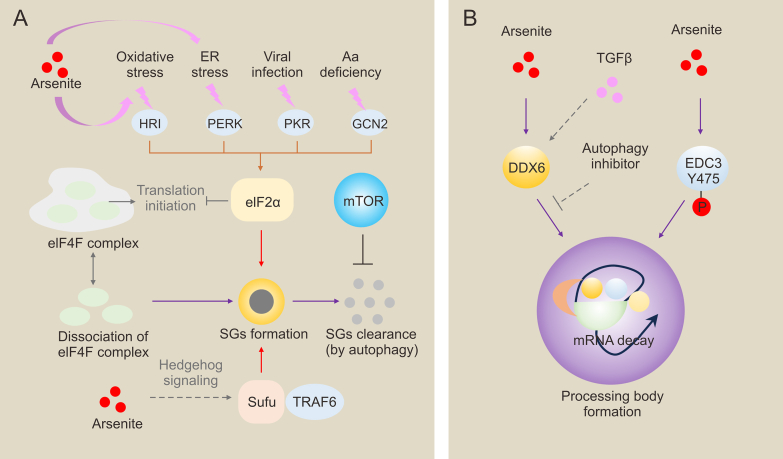
Arsenic compounds and membraneless organelles formations in the cytoplasm. (A) The mechanism of A_2_O_3_-induced stress granules (SGs). The eukaryotic translation initiation factor 4F (eIF4F) complex and hedgehog pathway are involved in SGs formation. SGs can be cleared by autophagy. (B) The functions of arsenite on processing body formation. Transforming growth factor-β (TGF-β) removal results in the clearance of processing bodies. Arsenite-induced processing bodies are dependent on DEAD-box helicase 6 (DDX6) and the phosphorylation of Enhancer of decapping 3 (EDC3) at Y475. ER: endoplasmic reticulum; HRI: heme-regulated inhibitor; PERK: RNA-dependent protein kinase (PKR)-like ER kinase; PKR: protein kinase R; GCN2: general control nonderepressible 2; mTOR: mammalian target of rapamycin; Sufu: Suppressor of fused homolog; TRAF6: TNF receptor associated factor 6.

Arsenite, a commonly used oxidative stress stressor in the laboratory, induces SG assembly in an eIF2α phosphorylation-dependent manner through reactive oxygen species (ROS) produced by its metabolism, and this induced effect is mediated by increasing eIF2α phosphorylation levels by multiple phosphokinases (HRI, PERK, PKR). Arsenite stress can decrease cellular sensitivity to arsenite, accompanied by altered SG kinetics. These arsenite-tolerant cells also showed differential changes in sensitivity to other chemotherapeutic agents [195]. In addition, ATO can affect autophagy. The increase in autophagosomes induced by arsenic exposure is due to increased arsenic-induced autophagy formation; Equally, arsenic inhibits the degradation of mature autophagosomes, and arsenicals-mediated autophagy may be through the AKT/mTOR signaling pathway [196,197]. In addition, arsenic can modulate other signaling pathways to affect the formation of SGs. Arsenide acts as an inhibitor of hedgehog signaling while suppressing hedgehog signaling is accompanied by stress granule formation [147]. Recent studies have shown that SUFU, the negative regulator of the hedgehog pathway, recruits TRAF6 to undergo a phase separation (Fig. 5A) [198]. Overall, arsenicals can induce the formation of stress granules, which may further exacerbate tumorigenesis and affect the efficacy of oncology drugs.

### 4.5 Arsenic compounds and other membraneless organelles

Arsenicals can also induce P-bodies in cytoplasm except for SG. P-bodies are membraneless organelles in the cytoplasm that contain various functional proteins and RNAs and are responsible for the degradation and storage of mRNAs [199]. Eystathioy et al. [200] used patient sera to build a cDNA library by immunoscreening, and a protein of 182 kD (named GW182) that interacts with mRNAs was identified and cloned from this library. GW182 is located in a separate region in the cytoplasm, which is different from any other known organelle, and this cytoplasmic region was named GW body (GWB). Sheth et al. [201] found that factors involved in mRNA degradation in yeast cells were clustered in a particular district of the cytoplasm along with mRNA degradation intermediates, which were named P-bodies. There is sufficient evidence that mammalian GWB proteins and yeast P-body proteins are homologous. P-bodies act as sites of mRNA degradation or translational repression, and it has been shown that Edc3, Pat1, and Lsm4 are essential for the assembly of P-bodies [202,203]. For the pathological significance of P-bodies, recent studies suggest that P-bodies are associated with the differentiation of epithelial-mesenchymal transition (EMT) and cancer stem cells (CSCs). Transforming growth factor-β (TGF-β) causes in mammary epithelial cells, along with the formation of P-bodies. In contrast, TGF-β removal results in the clearance of P-bodies. However, autophagy inhibitors can enhance P-bodies clearance and block EMT [204]. DEAD-box helicase 6 (DDX6), a protein essential for P-body assembly, blocks P-body formation by disrupting the DDX6 gene, which prevents EMT and metastasis *in vivo*. Lsm1, one of the components of P-bodies, is involved in mRNA degradation, which mediates EMT, chemo-resistance, and metastasis in pancreatic cancer cells and prostate cancer [205,206]. Arsenide-induced P-bodies are dependent on DDX6, which knockdown can reverse arsenide-induced P-bodies formation (Fig. 5B) [207]. In addition, recent studies also found that EDC3 regulates P-bodies responses to arsenic by controlling protein-protein interactions through phosphorylation at Y475 (Fig. 5B) [152].

In addition to the actions of ATO on the membraneless organelles mentioned above, ATO may also be involved in the association between several other membraneless organelles. FLASH is a hugely multifunctional protein, and previous studies have shown that FLASH localizes to the Cajal vesicle, PML-NBs, and the histone locus bodies [208,209]. However, ATO triggers the movement of FLASH to the PML Body recruitment and is accompanied by the degradation of FLASH protein [208]. With more functional studies on various membraneless organelle components, we may further expand the targets of ATO treatment in the future.

## 5 Conclusion and perspective

The role and mechanism of ATO in APL have been widely recognized, and multiple studies in vivo have confirmed that ATO also has a tumor-killing effect in other tumors. However, the toxic effects of ATO have always constrained its drug development, and researchers lacked an understanding of its toxicity at the organelle level. In recent years, with the deepening study of phase separation and membraneless organelles, more and more experimental evidence suggests that ATO plays a significant role in various membraneless organelles’ formation, which may be crucial in determining their pharmacological and toxic effects. Here, this review systematically summarizes the effects of arsenic compounds on different membraneless organelles. On the one hand, ATO can play a therapeutic role in APL by reducing the formation of PML-NBs; long-term ATO treatment can induce ARS2 expression and inhibit paraspeckles formation, which may play a significant role in relapsed ALL. On the other hand, arsenite can promote the formation of nuclear speckles, SGs, processing bodies, etc. These membraneless organelles exacerbate tumorigenesis and lead to drug resistance, which may be the mechanism of arsenic carcinogenesis.

Nowadays, the strategy for arsenic compounds’ application is to enhance efficacy and reduce toxicity. However, the methods to reduce the toxicity of arsenic compounds have always been a problem. Given the differences in the effects of arsenide on different membraneless organelles, we can combine it with other chemicals to enhance its functions on PML-NB and inhibit the formation of other membraneless organelles that promote tumor development. Due to the role of hedgehog in the formation of SGs, combining hedgehog inhibitors and arsenic compounds may further improve the efficacy of arsenic compounds while reducing the formation of SGs. Meanwhile, SGs can be cleared by autophagy. Enhancing autophagy may also be one method to decrease the toxicity of arsenic compounds. ATO-ATRA combination can further improve its efficacy in AML treatment. Some studies have also pointed out that ATRA can induce autophagy, which may help to clear SGs. Autophagy activators can enhance P-body clearance and block EMT. Therefore, autophagy inducers and ATO combination may improve efficacy and reduce the toxicity of arsenic compounds. In addition, Proteolysis-targeting chimera functions as a potential technology for targeted protein degradation, which may also increase the efficiency and reduce the toxicity of arsenic compounds. The development of targeted degradation agents based on key components of membraneless organelles may further decrease the toxic reactions of arsenic compounds.

However, there may be differences in the response of intracellular membraneless organelles to arsenic compounds treatment in different tumors due to the heterogeneity of tumors and the tumor microenvironment. Similarly, the concentrations and time of action of arsenic compounds are also significant for the functions of membraneless organelles. Targeting pathological phase separation accurately in various cells still requires a deeper understanding of phase separation and membraneless organelles. With a further understanding of membraneless organelles, we expect the development of arsenic to target specific membraneless organelles for tumor therapy, which may exert therapeutic efficacy and avoid the oncogenic effects of some membraneless organelles.

## CRediT author statement

**Meiyu Qu**: Validation, Formal analysis, Investigation, Data curation, Writing - Original draft preparation, Reviewing and Editing; **Qiangqiang He**: Formal analysis, Investigation, Data curation, Writing - Original draft preparation, Reviewing and Editing; **Hangyang Bao**: Investigation, Data curation; **Xing Ji**: Investigation, Data curation; **Tingyu Shen**: Writing - Reviewing and Editing; **Muhammad Qasim Barkat**: Writing - Reviewing and Editing; **Ximei Wu**: Resources, Writing - Reviewing and Editing, Supervision, Funding acquisition; **Linghui Zeng**: Resources, Writing - Reviewing and Editing, Supervision, Funding acquisition.

## Declaration of competing interest

The authors declare that there are no conflicts of interest.
